# Supplementary material for: Changes of Material Elastic Properties during Healing of Ruptured Achilles Tendons Measured with Shear Wave Elastography: A Pilot Study
Source: Int J Mol Sci. 2020 May 12;21(10):3427. doi: 10.3390/ijms21103427 (PMC7279368; doi:10.3390/ijms21103427)
Supplement: Supplementary file 1 [file ijms-21-03427-s001.pdf]

Patient Data*

Shear wave velocity (mean in [m/s])

Elastographic behaviour on a time scale up to one year

Patient # 1

A: 48
S: male
B: 29.5
M: bike accident
D: 3.4 cm
G: 0.8 cm
C: -
T: O

time point	Injured Tendon				Contral site	
	Rupture area	Distal area	Proximal area	Insertion	Tendon	Insertion
0 w	5.05	7.37	7.40	9.92	9.98	9.99
1 w	7.76	4.48	4.55	8.49		
3 w	6.23	4.46	4.58	8.56	9.84	9.43
6 w	6.96	5.41	4.97	9.60		
9 w						
12 w		10.00	8.72	9.98	9.55	8.98
6 m	6.75		6.78	7.45	9.94	9.06
9 m	9.99	10.00	10.00	9.80	9.93	9.51
12 m	10.00	9.84	9.98	10.00	9.70	9.63

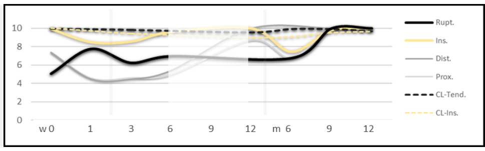

Patient # 1

Patient # 2

A: 52
S: male
B: -
M: during physiotherapeutical practice after ACL rupture
D: 3.7 cm
G: 1.8 cm
C: smoking
T: O

time point	Injured Tendon				Contral site	
	Rupture area	Distal area	Proximal area	Insertion	Tendon	Insertion
0 w	3.36	4.02	7.13	6.44	9.97	9.43
1 w	5.37	4.87	5.44	7.48		
3 w	5.98	5.20	5.61	6.06	9.95	9.64
6 w		9.99	10.00	9.99		
9 w	10.00	9.98	9.28	9.61		
12 w	9.97	9.97	9.43	9.95	9.27	9.45
6 m	9.98	9.94	8.86	9.96	9.92	9.87
9 m	10.00	10.00	9.99	10.00	10.00	9.63
12 m	8.52	9.66	9.96	10.00	9.81	9.97

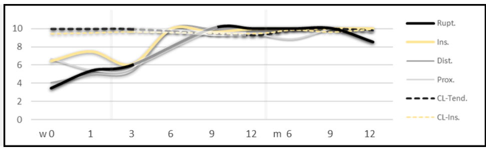

Patient # 2

Patient # 3

A: 29
S: male
B: 24.7
M: tennis
D: 5.6 cm
G: 1.3 cm
C: smoking
T: NO

time point	Injured Tendon				Contral site	
	Rupture area	Distal area	Proximal area	Insertion	Tendon	Insertion
0 w	6.40	6.07	3.83	9.93	9.74	9.82
1 w						
3 w	4.36	6.30	7.22	8.59	9.97	10.00
6 w	8.92	9.37	9.05	9.58		
9 w	6.79	6.32	8.54	8.32		
12 w	7.60	6.41	8.63	7.62	8.58	8.47
6 m	9.05	6.76	9.77	6.93	7.67	9.14
9 m	9.99	10.00	9.54	9.45	9.88	9.63
12 m	9.66	9.68	9.97	9.82	9.97	9.69

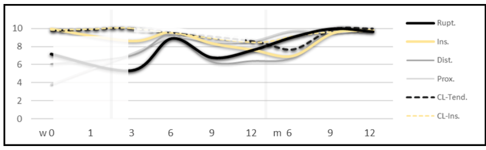

Patient # 3

Patient # 4

P: 4
A: 21
S: male
B: 20.4
M: soccer
D: 5.6 cm
G: 0.8 cm
C: -
T: NO

time point	Injured Tendon				Contral site	
	Rupture area	Distal area	Proximal area	Insertion	Tendon	Insertion
0 w	3.06	7.98	4.19	9.86	9.92	10.00
1 w	2.95	4.66	3.72	9.17		
3 w	8.00	9.59	8.85	8.93	9.94	9.98
6 w	9.36	7.05	6.93	8.56		
9 w	7.96	6.89	6.82	9.15		
12 w	9.46		8.00	6.39	8.11	8.11
6 m	9.97	9.33	9.45	9.80	9.31	9.96
9 m	9.68	9.54	8.93	9.49	9.34	10.00
12 m	8.39	9.64	9.51	9.87	9.53	10.00

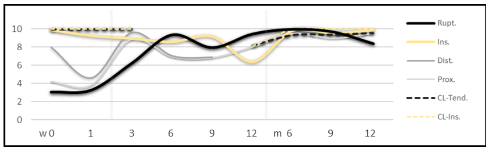

Patient # 4

Patient # 5

A: 29
S: male
B: 34.9
M: soccer
D: 4.4 cm
G: 0.9 cm
C: -
T: NO

time point	Injured Tendon				Contral site	
	Rupture area	Distal area	Proximal area	Insertion	Tendon	Insertion
0 w	5.03	5.19	4.16	9.65	9.92	10.00
1 w	3.54	3.24	3.21	5.68		
3 w	4.64	3.68	4.66	4.51	9.65	9.76
6 w	9.20	8.47	8.55	9.06		
9 w	8.66	9.61	8.65	9.19		
12 w	10.00	8.32	8.32	7.72	9.98	9.85
6 m						
9 m	9.94	9.88	9.98	8.33	9.53	9.66
12 m	9.97	9.32	7.55	8.68	9.53	9.86

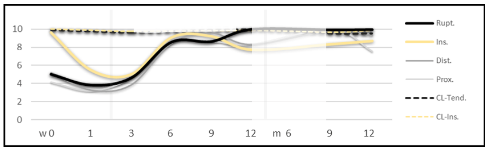

Patient # 5

Patient # 6

A: 26
S: male
B: 21.1
M: ankle hyperextension during fall while bouldering
D: 4.9 cm
G: 0.3 cm
C: -
T: NO

time point	Injured Tendon				Contral site	
	Rupture area	Distal area	Proximal area	Insertion	Tendon	Insertion
0 w	3.00	3.34	3.57	8.71	8.53	9.99
1 w	3.06	3.01	3.01	8.19		
3 w	3.94	5.19	3.26	7.23	7.03	7.47
6 w	9.43	9.54	8.35	8.93		
9 w	9.96	9.36	9.99	8.73		
12 w	9.86	9.24	10.00	9.13	8.50	9.99
6 m	9.95	9.69	9.89	9.41	9.67	9.82
9 m	9.87	9.68	9.54	9.96	9.59	9.51
12 m	9.51	9.10	7.02	8.13	9.48	9.90

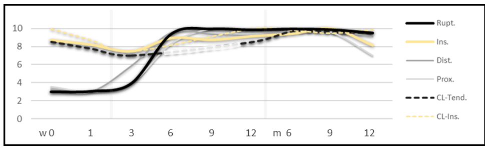

Patient # 6

Patient # 7

A: 62
S: male
B: 37.5
M: step on doorsill
D: 4.9 cm
G: 0.5 cm
C: diabetes, polyneuropathy, pAOD
T: NO

time point	Injured Tendon				Contral site	
	Rupture area	Distal area	Proximal area	Insertion	Tendon	Insertion
0 w	3.74	3.10	4.14	7.44	9.92	9.88
1 w	3.42	3.39	5.21	8.34		
3 w	4.45	5.34	7.32	7.88		
6 w	7.22	6.93	4.64	5.89		
9 w	7.67	8.16	6.44	8.86		
12 w	7.79	7.97	6.92	9.28	5.34	9.97
6 m	9.88	9.99	9.46	9.98	7.24	8.61
9 m	9.80	10.00	8.44	9.77	9.44	9.80
12 m	9.94	9.99	8.82	9.47	8.61	9.79

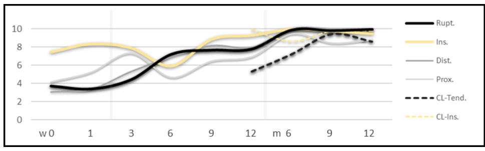

Patient # 7

Patient # 8

A: 44
S: male
B: 25.3
M: badminton
D: 5.3 cm
G: 0.3 cm
C: -
T: NO

time point	Injured Tendon				Contral site	
	Rupture area	Distal area	Proximal area	Insertion	Tendon	Insertion
0 w		4.71	5.68	9.81	9.93	9.97
1 w	4.14	3.98	3.90	5.81		
3 w	4.03	4.05	3.65	5.90		
6 w	7.25	6.82	5.89	8.87		
9 w	8.67	9.31	8.60	10.00		
12 w	9.63	8.62	6.21	8.72	9.21	9.99
6 m	9.91	8.78	7.90	8.78	9.95	9.73
9 m	9.90	9.00	8.68	9.38	9.61	9.96
12 m	10.00	9.89	8.85	9.88	9.56	9.48

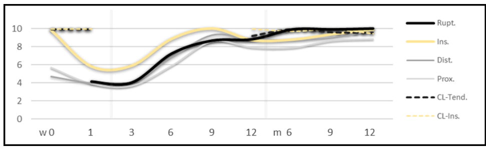

Patient # 8

Patient # 9

A: 32
S: male
B: 25
M: soccer
D: 5.3 cm
G: 0 cm
C: smoking
T: O

time point	Injured Tendon				Contral site	
	Rupture area	Distal area	Proximal area	Insertion	Tendon	Insertion
0 w	3.00	3.84	3.08	7.37	9.89	9.85
1 w	3.02	3.20	3.33	8.66		
3 w	5.05	4.09	5.81	6.70	7.66	9.22
6 w	8.96	7.03	8.01	7.91		
9 w	9.47	9.70	9.33	9.74		
12 w	10.00	9.99	9.36	9.67	9.86	9.00
6 m	10.00	9.93	9.10	9.95	9.85	9.93
9 m	9.96	10.00	8.13	9.94	9.11	9.89
12 m	9.98	10.00	8.68	9.96	9.91	9.10

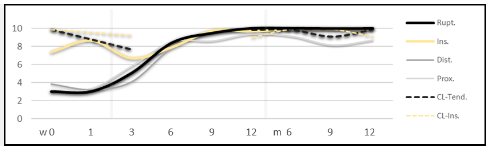

Patient # 9

Patient # 10

A: 39
S: M
B: 25.9
M: soccer
D: 3.7 cm
G: 0.3 cm
C: -
T: NO

time point	Injured Tendon				Contral site	
	Rupture area	Distal area	Proximal area	Insertion	Tendon	Insertion
0 w	3.04	4.99	4.10	9.88	9.05	9.81
1 w	5.88	6.45	4.77	8.98		
3 w	9.31	8.94	7.09	9.36	9.60	10.00
6 w	8.97	7.90	5.25	8.84		
9 w						
12 w	9.31	7.66	6.51	9.61	8.46	10.00
6 m	9.42	9.42	9.32	9.37	9.94	9.62
9 m	9.99	9.98	8.34	9.79	9.77	10.00
12 m	9.04	8.21	6.63	9.39	9.15	9.00

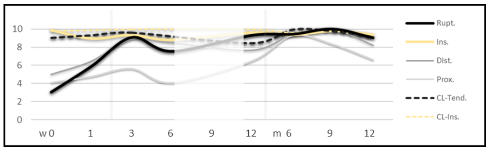

Patient # 10

Patient # 11

A: 48
S: female
B: 35.2
M: ballroom dancing
D: n.a.
G: 0 cm
C: -
T: NO

time point	Injured Tendon				Contral site	
	Rupture area	Distal area	Proximal area	Insertion	Tendon	Insertion
0 w						
1 w	3.74	4.08	4.59	9.18		
3 w	6.36	5.39	4.61	8.34	7.77	8.89
6 w	9.71	9.53	5.38	9.99		
9 w						
12 w	9.30	7.30	9.72	9.10	7.97	8.68
6 m	9.98	8.81	8.47	9.93	8.35	9.93
9 m	9.97	9.78	9.13	9.22	9.75	8.01
12 m	10.00	9.80	8.64	9.56	9.94	9.81

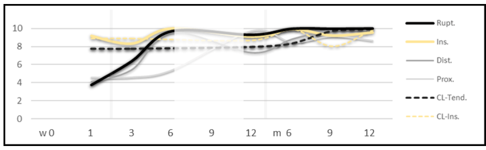

Patient # 11

Patient # 12

A: 45
S: male
B: n.a.
M: climbig down the ladder
D: 3.4 cm
G: 3 cm
C: smoking
T: NO

time point	Injured Tendon				Contral site	
	Rupture area	Distal area	Proximal area	Insertion	Tendon	Insertion
0 w	3.17	3.07	3.09	9.78	9.14	9.49
1 w	3.74	4.08	4.59	9.18		
3 w						
6 w	4.60	9.28	5.82	10.00		
9 w						
12 w	8.63	9.10	7.43	9.39		
6 m						
9 m	7.62	8.81	7.98	8.21	8.75	9.48
12 m						

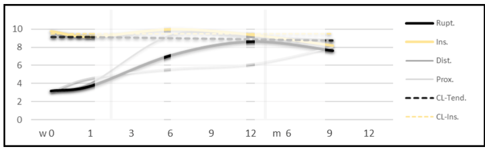

Patient # 12

Supplemented Data: Demographics, Shear wave velocities and elastographic curves of the included patients

* A: Age; S: Sex; B: BMI; M: Trauma mechanism; D: Distance of rupture from calcaneus; G: Lenght of gap in 0°; C: Comorbidities; T: Treatment (NO = non-operative, O = operative (grey background))
